# Free Vibration Analysis of Moderately Thick Orthotropic Functionally Graded Plates with General Boundary Restraints

**DOI:** 10.3390/ma11020273

**Published:** 2018-02-09

**Authors:** Yu Fu, Jianjun Yao, Zhenshuai Wan, Gang Zhao

**Affiliations:** Institute of Intelligent Manufacturing and Robotics, College of Mechanical and Electrical Engineering, Harbin Engineering University, Harbin 150001, China; travisyao@126.com (J.Y.); 18242311944@163.com (Z.W.); 18846440358@163.com (G.Z.)

**Keywords:** orthotropic functionally graded plates, modified Fourier series method, free vibration, general boundary restraints, gradient index, volume fraction

## Abstract

In this paper, a modified Fourier series method is presented for the free vibration of moderately thick orthotropic functionally graded plates with general boundary restraints based on the first-order shear deformation theory. Regardless of boundary restraints, displacements and rotations of each plate are described as an improved form of double Fourier cosine series and several closed-form auxiliary functions to eliminate all the boundary discontinuities and jumps. Exact solutions are obtained by the energy functions of the plates based on Rayleigh-Ritz method. The convergence and reliability of the current method and the corresponding theoretical formulations are verified by comparing the present results with those available in the literature, and numerous new results for orthotropic functionally graded (OFG) plates with general boundary restraints are presented. In addition, the effects of gradient index, volume fraction and geometric parameters on frequencies with general boundary restraints are illustrated.

## 1. Introduction

As a kind of novel composite materials, functionally graded materials (FGMs) can be characterized by the variation in composition and structure gradually over volume, resulting in continuous changes along the desired directions. Compared to laminated plates, the continuity of FGMs properties eliminates interfacial stresses at the junctions of materials [1,2,3,4]. Therefore, FG plates have been widely used in various engineering fields, such as aircraft, nuclear and automobile manufacturing [5,6,7,8,9]. As we all known, dynamic load is unavoidable on the practical applications, and it may lead to fatigue damage and stability reduction of the structures. Thus, it is necessary to study the vibration characteristics of FG plate structures.

To deal with the vibration problem of FG plates, many accurate and efficient calculation methods have been developed in the last few decades, such as extended Kantorovich method [10], Ritz method [11,12,13], power series method [14], meshless method [15,16,17], wave propagation approach [18], finite element method [19,20], etc. Chi et al. [21,22] studied the bending problem of FG rectangular plates based on the classical plate theory. Qian et al. [23] applied high-order shear and normal deformable plate theory to study the static and dynamic deformations of FG plates. Liu et al. [24] used an element-free Galerkin method to study the dynamic response of FG plate containing distributed piezoelectric actuators and sensors. It should be emphasized that most of these methods were applied to isotropic structures which are only considering the change of the Young’s modulus in the thickness. However, due to the limitations of the process conditions, most of the FGMs are orthotropic. The vibration analysis of the orthotropic functionally graded (OFG) plates has been the goal of intensive research, and many studies have been devoted to the OFG plates in the literature. Ramirez et al. [25] used the discrete layer theory in combination with the Ritz method to obtain an approximate solution for static analysis of OFG plates. Zhang et al. [26] adapted the third-order shear deformation theory to analyze chaotic vibrations of an OFG plate. Huang et al. [27] developed a discrete method for solving the vibration problem of orthotropic rectangular plates with variable thickness in one or two directions. Although these methods give sufficiently accurate results for thin plates, they are not valid for the vibration analysis of the moderately thick plates.

To eliminate the deficiency of the aforementioned methods, a Fourier series method was presented by Li [28,29]. This method has been subsequently transferred to the vibration analysis of more structures with various restraints [30,31,32,33,34,35,36,37]. From the review of the literature, most of the previous studies on the OFG plates are defined in a single volume distribution type. However, there are a variety of possible volume distributions in practical engineering applications, and these distributions have great influence on the vibration properties of the OFG plates. According to the effects of gradient index on the volume fraction in different distributions, the material properties change continuously through the thickness of the OFG plates.

Therefore, the objective of the present work is to provide an accurate and reliable method for the free vibration analysis of moderately thick OFG plates in various volume distribution types with general restraints. Displacements and rotations of each plate, regardless of boundary restraints, are described as an improved form of double Fourier cosine series and several closed-form auxiliary functions. Exact solutions are obtained by the energy functions of the plates based on Rayleigh–Ritz method. The excellent accuracy and reliability of the current results are verified by comparing the present solutions with those available in the literature. Studies focused on free vibration properties of OFG plates are presented, which may serve as a supplement of the material performance of OFG plates.

## 2. Theoretical Formulation

### 2.1. Model Description

**Figure 1:**
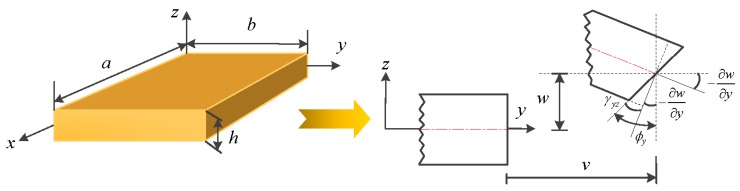
Schematic diagram of a moderately thick OFG plate with the undeformed and deformed geometries of an edge including shear deformation.

A moderately thick OFG plate with length *a*, width *b*, and uniform thickness *h* is depicted in Figure 1. The reference surface is taken to be its middle surface where the plate geometry and dimensions are arranged in a Cartesian coordinate system (*x*, *y*, *z*). The displacements of the plate in the *x*, *y*, and *z* directions are denoted by *u*, *v* and *w*, respectively. The general boundary conditions are assumed to be restrained by three independent springs (translational, rotational and torsional springs) placed at the ends. Assigning the stiffness of the springs with various values from zero to infinity is equivalent to imposing different boundary forces on the plate. For example, a free boundary is obtained by setting the stiffness of springs to zero, and a clamped boundary is obtained by setting the stiffness of springs to infinity. For moderately thick plates, the Kirchhoff hypothesis is relaxed by assuming that the normal to the undeformed middle surface is not perpendicular to the deformed middle surface.

### 2.2. Material Properties

Typically, OFG plates are made from a mixture of two materials in different proportions, for example, the metal and ceramic used in the following analyses are listed in Table 1. Material parameters per unit volume are assumed to vary continuously through the plate thickness and can be obtained:

(1) $$\begin{array}{c} E_{1}=E_{f}V_{f}+E_{m}V_{m}\\ E_{2}=\frac{E_{f}E_{m}}{E_{f}V_{m}+E_{m}V_{f}}\\ \rho =\rho_{f}V_{f}+\rho_{m}V_{m}\\ \nu_{12}=\nu_{f}V_{f}+\nu_{m}V_{m} \end{array}$$

where *E*_1_ and *E*_2_ represent the horizontal and vertical Young’s modulus, respectively; $\nu_{12}$ and *ρ* are the major Poisson ratio and density, respectively; $E_{f}$ and $E_{m}$ are the Young’s modulus of ceramic and metal, respectively; $\nu_{f}$ and $\rho_{f}$ are ceramic’s Poisson ratio and density, respectively; $V_{m}$ and $\rho_{m}$ are metal’s Poisson ratio and density, respectively; and $V_{f}$ and $V_{m}$ denote the volume fractions of ceramic and metal, respectively. The shear modulus of the material can be given by:

(2) $$\begin{array}{c} G_{f}=\frac{E_{f}}{2(1+v_{f})},\;\;\;\;\;\;\;G_{m}=\frac{E_{m}}{2(1+v_{m})}\\ G_{12}=G_{13}=G_{23}=\frac{G_{f}G_{m}}{G_{f}V_{m}+G_{m}V_{f}} \end{array}$$

where $G_{f}$ and $G_{m}$ are the shear modulus of ceramic and metal. *G*_12_ is composite structure’s shear modulus. Furthermore, according to different ceramic-to-metal volume distributions in the thickness direction, the OFG plates are assumed as three types, P-, C- and S-OFG, respectively. $V_{f}$ in the thickness direction *z* can be expressed as:

(3) $$P:\;\;\;\;V_{f}=V_{1}+(V_{2}-V_{1}){\left(\frac{1}{2}+\frac{z}{h}\right)}^{p}\;C:\;\;\;\;V_{f}=V_{1}+(V_{2}-V_{1}){\left(\frac{1}{2}+\frac{z}{h}+{\left(\frac{1}{2}-\frac{z}{h}\right)}^{2}\right)}^{p}\;S:\;\;\;\;V_{f}=\{\begin{array}{c} \frac{V_{1}}{2}{\left(1-\frac{2z}{h}\right)}^{p}+V_{2}\left(1-\frac{1}{2}{\left(1-\frac{2z}{h}\right)}^{p}\right),\;\;0\leq z\leq \frac{h}{2}\\ V_{1}\left(1-\frac{1}{2}{\left(1+\frac{2z}{h}\right)}^{p}\right)+\frac{V_{2}}{2}{\left(1+\frac{2z}{h}\right)}^{p},\;\;-\frac{h}{2}\leq z\leq 0 \end{array}$$

where $V_{1}$ and $V_{2}$ are available minimum and maximum values of $V_{f}$. Especially for P- and C-OFG, $V_{1}$ and $V_{2}$ represent the ceramic volume fractions of bottom and top surfaces, respectively. In addition, *p* is the gradient index and only takes non-negative values. When the value of *p* varies between zero and infinity, non-homogeneous material properties can be obtained. 

### 2.3. Stress–Strain Relations and Stress Resultants

Based on the assumptions of the first-order shear deformation theory (FSDT) [38,39], the displacement components of moderately thick OFG plates are:

(4) $$\begin{array}{c} U(x,y,z)=u(x,y)+z\phi_{x}(x,y)\\ V(x,y,z)=v(x,y)+z\phi_{y}(x,y)\\ W(x,y,z)=w(x,y) \end{array}$$

where *u*, *v* and *w* denote the middle surface displacements of the plate in the *x*, *y* and *z* directions, respectively. *ϕ_x_* and *ϕ_y_* represent the transverse normal rotations of the reference surface respect to the *y* and *x* directions. Under the assumption of linear and small deformation, the strains and curvature can be defined in terms of displacements as:

(5) $$\left\{\begin{array}{c} \varepsilon_{x}\\ \varepsilon_{y}\\ \gamma_{xy} \end{array}\right\}=\left\{\begin{array}{c} \varepsilon_{x}^{0}+z\chi_{x}\\ \varepsilon_{y}^{0}+z\chi_{y}\\ \gamma_{xy}+z\chi_{xy} \end{array}\right\}=\left\{\begin{array}{c} \frac{\partial u}{\partial x}\\ \frac{\partial v}{\partial y}\\ \frac{\partial v}{\partial y}+\frac{\partial v}{\partial x} \end{array}\right\}+z\left\{\begin{array}{c} \frac{\partial \phi_{x}}{\partial x}\\ \frac{\partial \phi_{y}}{\partial y}\\ \frac{\partial \phi_{x}}{\partial y}+\frac{\partial \phi_{y}}{\partial x} \end{array}\right\}$$

(6) $$\left\{\begin{array}{c} \gamma_{xz}\\ \gamma_{yz} \end{array}\right\}=\left\{\begin{array}{c} \gamma_{{}_{xz}}^{0}\\ \gamma_{{}_{yz}}^{0} \end{array}\right\}=\left\{\begin{array}{c} \frac{\partial u}{\partial z}+\frac{\partial w}{\partial x}\\ \frac{\partial v}{\partial z}+\frac{\partial w}{\partial y} \end{array}\right\}=\left\{\begin{array}{c} \phi_{x}+\frac{\partial w}{\partial x}\\ \phi_{y}+\frac{\partial w}{\partial y} \end{array}\right\}$$

where $\varepsilon_{x}$, $\varepsilon_{y}$ and $\gamma_{xy}$ are the normal and shear strains in the *x*, *y* and *z* directions. $\gamma_{xz}$ and $\gamma_{yz}$ indicate the transverse shear strains, which are assumed to be constants through the thickness. The matrix can be denoted as:

(7) $$\begin{array}{c} \varepsilon =(\frac{\partial u}{\partial x},\frac{\partial v}{\partial y},\frac{\partial u}{\partial y}+\frac{\partial v}{\partial x}){}^{T}\\ \chi =(\frac{\partial \phi_{x}}{\partial x},\frac{\partial \phi_{y}}{\partial y},\frac{\partial \phi_{x}}{\partial y}+\frac{\partial \phi_{y}}{\partial x}){}^{T}\\ \gamma =\left(\frac{\partial u}{\partial z}+\frac{\partial w}{\partial x},\frac{\partial v}{\partial z}+\frac{\partial w}{\partial y}\right){}^{T}. \end{array}$$

According to the generalized Hooke’s law [40], the corresponding stress–strain relations of a moderately thick OFG plate can be expressed as follows:

(8) $$\left\{\begin{array}{c} \sigma_{x}\\ \sigma_{y}\\ \tau_{yz}\\ \tau_{xz}\\ \tau_{xy} \end{array}\right\}=\left[\begin{array}{ccccc} Q_{11}(z) & Q_{12}(z)\; & 0 & 0 & 0\\ Q_{12}\;(z)\;\; & Q_{22}(z)\; & 0 & 0 & 0\\ 0 & 0 & Q_{44}(z) & 0 & 0\\ 0 & 0 & 0 & \;Q_{55}(z)\; & 0\\ 0 & 0 & 0 & 0 & Q_{66}(z) \end{array}\right]\left\{\begin{array}{c} \varepsilon_{x}\\ \varepsilon_{y}\\ \gamma_{xz}\\ \gamma_{yz}\\ \gamma_{xy} \end{array}\right\}$$

where the elastic constants $Q_{ij}(z)$ are defined in terms of the material properties as:

(9) $$\begin{array}{c} Q_{11}(z)=\frac{E_{1}(z)}{1-v_{12}v_{21}}\;\;\;\;Q_{44}(z)=G_{23}(z)\\ Q_{22}(z)=\frac{E_{2}(z)}{1-v_{12}v_{21}}\;\;\;\;Q_{55}(z)=G_{13}(z)\\ Q_{12}(z)=\frac{E_{1}(z)v_{21}}{1-v_{12}v_{21}}\;\;\;\;Q_{66}(z)=G_{12}(z) \end{array}$$

The force and moment resultants are obtained by integrating the stresses over the plate thickness:

(10) $${\left(N_{x},N_{y},N_{xy}\right)}^{T}=\int_{-h/2}^{h/2}\left[\sigma_{x},\sigma_{y},\tau_{xy}\right]dz$$

(11) $${\left(M_{x},M_{y},M_{xy}\right)}^{T}=\int_{-h/2}^{h/2}\left[\sigma_{x},\sigma_{y},\tau_{xy}\right]zdz$$

(12) $${\left(Q_{x},Q_{y}\right)}^{T}=\int_{-h/2}^{h/2}\left[\tau_{xz},\tau_{yz}\right]dz$$

where $N_{x}$, $N_{y}$ and $N_{xy}$ are the force resultants. $M_{x}$, $M_{y}$ and $M_{xy}$ are the moment resultants. The transverse shear force resultants are denoted as $Q_{x}$ and $Q_{y}$, respectively. Performing the integration operation in Equations (10)–(12), the force and moment resultants can be written as:

(13) $$\left[\begin{array}{c} N_{x}\\ N_{y}\\ N_{xy} \end{array}\right]=\left[\begin{array}{ccc} A_{11} & A_{12} & 0\\ A_{12} & A_{22} & 0\\ 0 & 0 & A_{66} \end{array}\right]\varepsilon +\left[\begin{array}{ccc} B_{11} & B_{12} & 0\\ B_{12} & B_{22} & 0\\ 0 & 0 & B_{66} \end{array}\right]\chi$$

(14) $$\left[\begin{array}{c} M_{x}\\ M_{y}\\ M_{xy} \end{array}\right]=\left[\begin{array}{ccc} B_{11} & B_{12} & 0\\ B_{12} & B_{22} & 0\\ 0 & 0 & B_{66} \end{array}\right]\varepsilon +\left[\begin{array}{ccc} D_{11} & D_{12} & 0\\ D_{12} & D_{22} & 0\\ 0 & 0 & D_{66} \end{array}\right]\chi$$

(15) $$\left[\begin{array}{c} Q_{x}\\ Q_{y} \end{array}\right]=\left[\begin{array}{cc} A_{44} & 0\\ 0 & A_{55} \end{array}\right]\gamma$$

The stiffness coefficients $A_{ij}$, $B_{ij}$ and $D_{ij}$ are expressed as:

(16) $$\left(A_{ij},B_{ij},D_{ij}\right)=\int_{-h/2}^{h/2}Q_{ij}(z)(1,z,z^{2})dz$$

### 2.4. Energy Functions

In this subsection, the modified Fourier series version of Rayleigh–Ritz method is presented. In the Rayleigh–Ritz method, a displacement field associated with undetermined coefficients is assumed firstly, and substituted into the Lagrangian energy function [41]. Then, the undetermined coefficients in the displacement field can be obtained by finding the stationary value of the energy function, namely, minimizing the energy function with respect to the undetermined coefficients and making them equal to zero. Finally, a series of equations related to corresponding coefficients can be achieved and summed up in matrix form as a standard characteristic equation. The desired frequencies of the structure can be determined easily by solving the standard characteristic equation.

For free vibration analysis, the Lagrangian energy function of the plates can be simplified and written in terms of the strain energy and kinetic energy functions as:

(17) $$L=T-U_{s}-U_{sp}$$

The strain energy *U*_s_ of the moderately thick OFG plates during vibration can be defined in terms of the middle surface strains, curvature changes and stress resultants as:

(18) $$U_{s}=\frac{1}{2}\int_{0}^{a}\int_{0}^{b}\left\{N_{x}\varepsilon_{x}^{0}+N_{y}\varepsilon_{y}^{0}+N_{xy}\varepsilon_{xy}^{0}+M_{x}\chi_{x}+M_{y}\chi_{y}+M_{xy}\chi_{xy}+Q_{x}\gamma_{xz}^{0}+Q_{y}\gamma_{yz}^{0}\right\}dydx$$

Substituting Equations (5), (6), and (13)–(15) into Equation (18), the strain energy can be expressed in terms of displacements (*u*, *v*, *w*) and rotations components (*ϕ_x_*, *ϕ_y_*) as:

(19) $$U_{s}=\frac{1}{2}\int_{0}^{a}\int_{0}^{b}\{\begin{array}{c} A_{11}{\left(\frac{\partial u}{\partial x}\right)}^{2}+A_{22}{\left(\frac{\partial v}{\partial y}\right)}^{2}+A_{44}{\left(\frac{\partial u}{\partial z}+\frac{\partial w}{\partial x}\right)}^{2}+A_{55}{\left(\frac{\partial v}{\partial z}+\frac{\partial w}{\partial y}\right)}^{2}+A_{66}{\left(\frac{\partial u}{\partial y}+\frac{\partial v}{\partial x}\right)}^{2}\\ +2A_{12}\left(\frac{\partial v}{\partial y}\right)\left(\frac{\partial u}{\partial x}\right)+2B_{11}\left(\frac{\partial u}{\partial x}\right)\left(\frac{\partial \phi_{x}}{\partial x}\right)+2B_{22}\left(\frac{\partial v}{\partial y}\right)\left(\frac{\partial \phi_{y}}{\partial y}\right)+2B_{12}\left(\frac{\partial u}{\partial x}\right)\left(\frac{\partial \phi_{x}}{\partial x}\right)\\ +2B_{12}\left(\frac{\partial v}{\partial y}\right)\left(\frac{\partial \phi_{x}}{\partial x}\right)+2B_{66}\left(\frac{\partial u}{\partial y}+\frac{\partial v}{\partial x}\right)\left(\frac{\partial \phi_{x}}{\partial y}+\frac{\partial \phi_{y}}{\partial x}\right)+D_{11}{\left(\frac{\partial \phi_{x}}{\partial x}\right)}^{2}+D_{22}{\left(\frac{\partial \phi_{y}}{\partial y}\right)}^{2}\\ +2D_{12}\left(\frac{\partial \phi_{x}}{\partial x}\right)\left(\frac{\partial \phi_{y}}{\partial y}\right)+D_{66}{\left(\frac{\partial \phi_{x}}{\partial y}+\frac{\partial \phi_{y}}{\partial x}\right)}^{2} \end{array}\}dydx$$

The kinetic energy *T* of the vibrating OFG plate is given by:

(20) $$T=\frac{1}{2}\int_{0}^{a}\int_{0}^{b}\left\{\begin{array}{c} I_{0}{\left(\frac{\partial u}{\partial t}\right)}^{2}+I_{0}{\left(\frac{\partial v}{\partial t}\right)}^{2}+I_{0}{\left(\frac{\partial w}{\partial t}\right)}^{2}+2I_{1}\left(\frac{\partial u}{\partial t}\right)\left(\frac{\partial \phi_{x}}{\partial t}\right)+2I_{1}\left(\frac{\partial v}{\partial t}\right)\left(\frac{\partial \phi_{y}}{\partial t}\right)\\ +I_{2}{\left(\frac{\partial \phi_{x}}{\partial t}\right)}^{2}+I_{2}{\left(\frac{\partial \phi_{y}}{\partial t}\right)}^{2} \end{array}\right\}dydx$$

Assuming the distributed external forces $q_{x}$, $q_{y}$ and $q_{z}$ are in the *x*, *y* and *z* directions, respectively. *m_x_* and *m_y_* are the external couples in the middle surface. Thus, the work *W_e_* done by the forces and moments is:

(21) $$W_{e}=\int_{0}^{a}\int_{0}^{b}\left\{q_{x}u+q_{y}v+q_{z}w+m_{x}\phi_{x}+m_{y}\phi_{y}\right\}\;dydx$$

**Figure 2:**
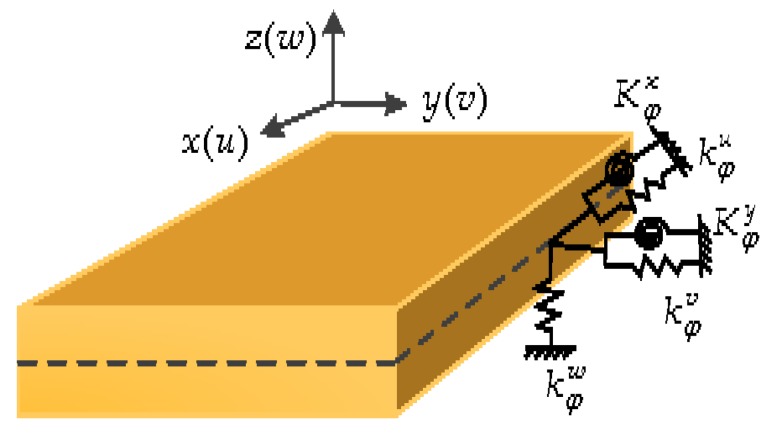
Boundary restraints of a moderately thick OFG plate.

$k_{\varphi }^{u}$, $k_{\varphi }^{v}$, $k_{\varphi }^{w}$, $K_{\varphi }^{x}$ and $K_{\varphi }^{y}$ ($\varphi =x_{0},x_{1},y_{0},y_{1}$) are used to indicate the rigidities (per unit length) of the boundary springs at the *x* = 0, *x* = *a*, *y* = 0 and *y* = b, respectively (see Figure 2). Therefore, the deformation strain energy (*U*_sp_) stored in the boundary springs can be expressed as:

(22) $$\begin{array}{cc} U_{sp} & =\frac{1}{2}\int_{0}^{a}\left\{{\left[k_{y_{0}}^{u}u^{2}+k_{y_{0}}^{v}v^{2}+k_{y_{0}}^{w}w^{2}+K_{y_{0}}^{x}\phi_{x}{}^{2}+K_{y_{0}}^{y}\phi_{y}{}^{2}\right]}_{y=0}+{\left[k_{y_{1}}^{u}u^{2}+k_{y_{1}}^{v}v^{2}+k_{y_{1}}^{w}w^{2}+K_{y_{0}}^{x}\phi_{x}{}^{2}+K_{y_{0}}^{y}\phi_{y}{}^{2}\right]}_{y=b}\right\}dx\\ & +\frac{1}{2}\int_{0}^{a}\left\{{\left[k_{x_{0}}^{u}u^{2}+k_{x_{0}}^{v}v^{2}+k_{x_{0}}^{w}w^{2}+K_{x_{0}}^{x}\phi_{x}{}^{2}+K_{x_{0}}^{y}\phi_{y}{}^{2}\right]}_{x=0}+{\left[k_{x_{1}}^{u}u^{2}+k_{x_{1}}^{v}v^{2}+k_{x_{1}}^{w}w^{2}+K_{x_{1}}^{x}\phi_{x}{}^{2}+K_{x_{1}}^{y}\phi_{y}{}^{2}\right]}_{x=a}\right\}dy \end{array}$$

### 2.5. Governing Equations and Boundary Restraints

By applying Hamilton’s principle, the governing equations of moderately thick OFG plates can be obtained:

(23) $$\frac{\partial N_{x}}{\partial x}+\frac{\partial N_{xy}}{\partial y}+q_{x}=I_{0}\frac{\partial^{2}u}{\partial t^{2}}+I_{1}\frac{\partial^{2}\phi_{x}}{\partial t^{2}}$$

(24) $$\frac{\partial N_{xy}}{\partial x}+\frac{\partial N_{y}}{\partial y}+q_{y}=I_{0}\frac{\partial^{2}v}{\partial t^{2}}+I_{1}\frac{\partial^{2}\phi_{y}}{\partial t^{2}}$$

(25) $$\frac{\partial Q_{x}}{\partial x}+\frac{\partial Q_{y}}{\partial y}+q_{z}=I_{0}\frac{\partial^{2}w}{\partial t^{2}}$$

(26) $$\frac{\partial M_{x}}{\partial x}+\frac{\partial M_{xy}}{\partial y}-Q_{x}+m_{x}=I_{1}\frac{\partial^{2}u}{\partial t^{2}}+I_{2}\frac{\partial^{2}\phi_{x}}{\partial t^{2}}$$

(27) $$\frac{\partial M_{xy}}{\partial x}+\frac{\partial M_{y}}{\partial y}-Q_{y}+m_{y}=I_{1}\frac{\partial^{2}v}{\partial t^{2}}+I_{2}\frac{\partial^{2}\phi_{y}}{\partial t^{2}}$$

The general boundary restraints for moderately thick OFG plates can be expressed as the following forms:

On *x* = 0

(28) $$N_{x}=k_{x_{0}}^{u}u,\;N_{xy}=k_{x_{0}}^{v}v,\;M_{x}=K_{x_{0}}^{x}\phi_{x},\;M_{xy}=K_{x_{0}}^{y}\phi_{y},\;Q_{x}=k_{x_{0}}^{w}w$$

On *x* = *a*

(29) $$N_{x}=-k_{x_{1}}^{u}u,\;N_{xy}=-k_{x_{1}}^{v}v,\;M_{x}=-K_{x_{1}}^{x}\phi_{x},\;M_{xy}=K_{x_{1}}^{y}\phi_{y},\;Q_{x}=-k_{x_{1}}^{w}w$$

On *y* = 0

(30) $$N_{xy}=k_{y_{0}}^{u}u,\;N_{y}=k_{y_{0}}^{v}v,\;M_{xy}=K_{y_{0}}^{x}\phi_{x},\;M_{y}=K_{y_{0}}^{y}\phi_{y},\;Q_{y}=k_{y_{0}}^{w}w$$

On *y* = b

(31) $$N_{xy}=-k_{y_{1}}^{u}u,\;N_{y}=-k_{y_{1}}^{v}v,\;M_{xy}=-K_{y_{1}}^{x}\phi_{x},\;M_{y}=-K_{y_{1}}^{y}\phi_{y},\;Q_{y}=-k_{y_{1}}^{w}w$$

### 2.6. Admissible Displacement Functions

In this subsection, we consider free vibration of moderately thick OFG plates with general boundary restraints. Although the Fourier functions exhibit an excellent numerical stability, conventional Fourier series expression will have a convergence problem along the boundary edges except for a few simple boundary restraints.

This article proposes a modified Fourier series method for the displacement and rotation components of the OFG plates, by an improved form of double Fourier cosine series and several closed-form auxiliary functions. Regardless of boundary conditions, each displacement and rotation component of the OFG plate is expanded as a modified Fourier series as:

(32) $$u(x,y)=\sum_{m=0}^{M}\sum_{n=0}^{N}A_{mn}\cos\lambda_{m}x\cos\lambda_{n}y+\sum_{l=1}^{2}\sum_{n=0}^{N}a_{l}^{n}\zeta_{l}^{a}(x)\cos\lambda_{n}y+\sum_{l=1}^{2}\sum_{m=0}^{M}b_{l}^{m}\zeta_{l}^{b}(y)\cos\lambda_{m}x$$

(33) $$v(x,y)=\sum_{m=0}^{M}\sum_{n=0}^{N}B_{mn}\cos\lambda_{m}x\cos\lambda_{n}y+\sum_{l=1}^{2}\sum_{n=0}^{N}c_{l}^{n}\zeta_{l}^{a}(x)\cos\lambda_{n}y+\sum_{l=1}^{2}\sum_{m=0}^{M}d_{l}^{m}\zeta_{l}^{b}(y)\cos\lambda_{m}x$$

(34) $$w(x,y)=\sum_{m=0}^{M}\sum_{n=0}^{N}C_{mn}\cos\lambda_{m}x\cos\lambda_{n}y+\sum_{l=1}^{2}\sum_{n=0}^{N}e_{l}^{n}\zeta_{l}^{a}(x)\cos\lambda_{n}y+\sum_{l=1}^{2}\sum_{m=0}^{M}f_{l}^{m}\zeta_{l}^{b}(y)\cos\lambda_{m}x$$

(35) $$\phi_{x}(x,y)=\sum_{m=0}^{M}\sum_{n=0}^{N}D_{mn}\cos\lambda_{m}x\cos\lambda_{n}y+\sum_{l=1}^{2}\sum_{n=0}^{N}g_{l}^{n}\zeta_{l}^{a}(x)\cos\lambda_{n}y+\sum_{l=1}^{2}\sum_{m=0}^{M}h_{l}^{m}\zeta_{l}^{b}(y)\cos\lambda_{m}x$$

(36) $$\phi_{y}(x,y)=\sum_{m=0}^{M}\sum_{n=0}^{N}E_{mn}\cos\lambda_{m}x\cos\lambda_{n}y+\sum_{l=1}^{2}\sum_{n=0}^{N}i_{l}^{n}\zeta_{l}^{a}(x)\cos\lambda_{n}y+\sum_{l=1}^{2}\sum_{m=0}^{M}j_{l}^{m}\zeta_{l}^{b}(y)\cos\lambda_{m}x$$

where $\lambda_{m}=m\pi /a$ and $\lambda_{n}=n\pi /b$. *M* and *N* denote the truncation numbers with respect to variables *x* and *y*, respectively. *A_mn_*, *B_mn_*, *C_mn_*, *D_mn_* and *E_mn_* are the Fourier expansion coefficients of the cosine Fourier series. $a_{l}^{n}$, $b_{l}^{m}$, $c_{l}^{n}$, $d_{l}^{m}$, $e_{l}^{n}$, $f_{l}^{m}$, $g_{l}^{n}$, $h_{l}^{m}$, $i_{l}^{n}$ and $j_{l}^{m}$ are the corresponding supplement coefficients. $\zeta_{l}^{a}(x)$ and $\zeta_{l}^{b}(y)$ denote the auxiliary polynomial functions introduced to remove all the discontinuities potentially associated with the first-order derivatives at the boundaries. The auxiliary functions are expressed as follows:

(37) $$\begin{array}{c} \zeta_{1}^{a}(x)=x{\left(\frac{x}{a}-1\right)}^{2}\;\;\zeta_{2}^{a}(x)=\frac{x^{2}}{a}\left(\frac{x}{a}-1\right)\\ \zeta_{1}^{b}(y)=y{\left(\frac{y}{b}-1\right)}^{2}\;\;\zeta_{2}^{b}(y)=\frac{y^{2}}{b}\left(\frac{y}{b}-1\right) \end{array}$$

It is easy to verify that,

(38) $$\begin{array}{c} \zeta_{1}^{a}(0)=\zeta_{1}^{a}(a)=\zeta_{1}^{a^{\prime }}(a)=0,\;\;\zeta_{1}^{a^{\prime }}(0)=1\\ \zeta_{2}^{a}(0)=\zeta_{2}^{a}(a)=\zeta_{2}^{a^{\prime }}(0)=0,\;\;\zeta_{2}^{a^{\prime }}(a)=1\\ \zeta_{1}^{b}(0)=\zeta_{1}^{b}(b)=\zeta_{1}^{b^{\prime }}(b)=0,\;\;\zeta_{1}^{b^{\prime }}(0)=1\\ \zeta_{2}^{b}(0)=\zeta_{2}^{b}(b)=\zeta_{2}^{b^{\prime }}(0)=0,\;\;\zeta_{2}^{b^{\prime }}(b)=1 \end{array}$$

All the expansion coefficients in Equation (25) can be treated independently and equally as the generalized coordinates and solved directly from the Ritz method. The method can be summed up in a matrix form as:

(39) $$(K-\omega^{2}M)G=0$$

where **K** and **M** is the stiffness matrix and mass matrix of the OFG plate, respectively. Both are symmetric matrices and can be written as:

(40) $$K=\left[\begin{array}{ccccc} K_{uu} & K_{uv} & K_{uw} & K_{u\phi_{x}} & K_{u\phi_{y}}\\ K_{uv} & K_{vv} & K_{vw} & Kv_{\phi_{x}} & K_{v\phi_{y}}\\ K_{uw} & K_{vw} & K_{ww} & K_{w\phi_{x}} & K_{w\phi_{y}}\\ K_{u\phi_{x}} & Kv_{\phi_{x}} & K_{w\phi_{x}} & K_{\phi_{x}\phi_{x}} & K_{\phi_{x}\phi_{y}}\\ K_{u\phi_{y}} & K_{v\phi_{y}} & K_{w\phi_{y}} & K_{\phi_{x}\phi_{y}} & K_{\phi_{y}\phi_{y}} \end{array}\right]$$

(41) $$M=\left[\begin{array}{ccccc} M_{uu} & 0 & 0 & M_{u\phi_{x}} & 0\\ 0 & M_{uu} & 0 & 0 & M_{v\phi_{y}}\\ 0 & 0 & M_{ww} & 0 & 0\\ M_{u\phi_{x}} & 0 & 0 & M_{\phi_{x}\phi_{x}} & 0\\ 0 & M_{v\phi_{y}} & 0 & 0 & M_{\phi_{y}\phi_{y}} \end{array}\right]$$

The coefficient eigenvector **G** is the unknown expansion coefficient in the series expansions, and determined for a given frequency, namely:

(42) $$G={\left[G^{u},G^{v},G^{w},G^{\phi_{x}},G^{\phi_{y}}\right]}^{T}$$

The Fourier coefficient eigenvector **G**, stiffness matrix **K** and mass matrix **M** are given in the Appendix A.

## 3. Numerical Results and Discussion

In this section, numerical examples for the free vibration analysis of moderately thick OPG plates with various gradient indexes and general boundary restraints are presented. Firstly, the convergence and reliability of the proposed modified Fourier series method is validated by comparing the current solutions with those results published in the literature under the distribution of P-OFG. Secondly, the free vibration behavior of OFG plates with general boundary restraints is studied. Then, the effects of gradient index *p* on the volume fraction in the thickness direction, in various distributions (P-, C- and S-OFG), are discussed. Finally, the relations between fundamental frequencies *f* (Hz) and gradient index *p* in the various volume fractions with general boundary restraints are contrasted and analyzed as well.

### 3.1. OFG Plates with General Boundary Restraints

The aforementioned general boundary restraints can be readily realized by assigning the stiffness of the boundary springs at proper values. Taking edge *x* = 0 as an example, the frequently encountered boundary restraints F (free edge), C (clamped edge) and S (simply-supported edge) can be defined as follows:

(43) $$\begin{array}{c} F:\;k_{x_{0}}^{u}=k_{x_{0}}^{v}=k_{x_{0}}^{w}=K_{x_{0}}^{x}=K_{x_{0}}^{y}=0\\ C:\;k_{x_{0}}^{u}=k_{x_{0}}^{v}=k_{x_{0}}^{w}=K_{x_{0}}^{x}=K_{x_{0}}^{y}=10^{7}D\\ S:\;k_{x_{0}}^{u}=k_{x_{0}}^{v}=k_{x_{0}}^{w}=K_{x_{0}}^{y}=10^{7}D,\;K_{x_{0}}^{x}=0 \end{array}$$

where $D=E_{1}h^{3}/12(1-v_{12}^{2})$ is the flexural stiffness of the plate. The accuracy and convergence of the present solution is demonstrated in Table 2 and Table 3. Table 2 compares the first five frequency parameters $\Omega =\omega a^{2}\sqrt{\rho h/D}$ of OFG plates with CCCC, SSSS and FFFF boundary restraints and thickness–length ratio a/b = 0.5. Three different thickness–length ratios, h/a = 0.1, 0.2 and 0.3, corresponding to the moderately thick plates, are considered in the comparison. The solution given by Jin et al. [35] by the three-dimensional elasticity method is provided for a direct comparison. The difference does not exceed 0.044% for the worst case, which is acceptable. 

The non-dimensional frequency parameters $\varpi =\omega h\sqrt{\rho_{m}/E_{m}}$ for square plates with CCCC, SSSS, CFCF, and SCSC boundary restraints are shown in Table 3. The thickness–length ratio used for the analysis is *h*/*a* = 0.2, and the truncation number is *M* = *N* = 9 and 11. It can be seen that the present solutions are in close agreement with the results obtained from TSDT method [15]. In general, a consistent agreement of the present results is seen from the tables by comparing with those available in the literature.

**Figure 3:**
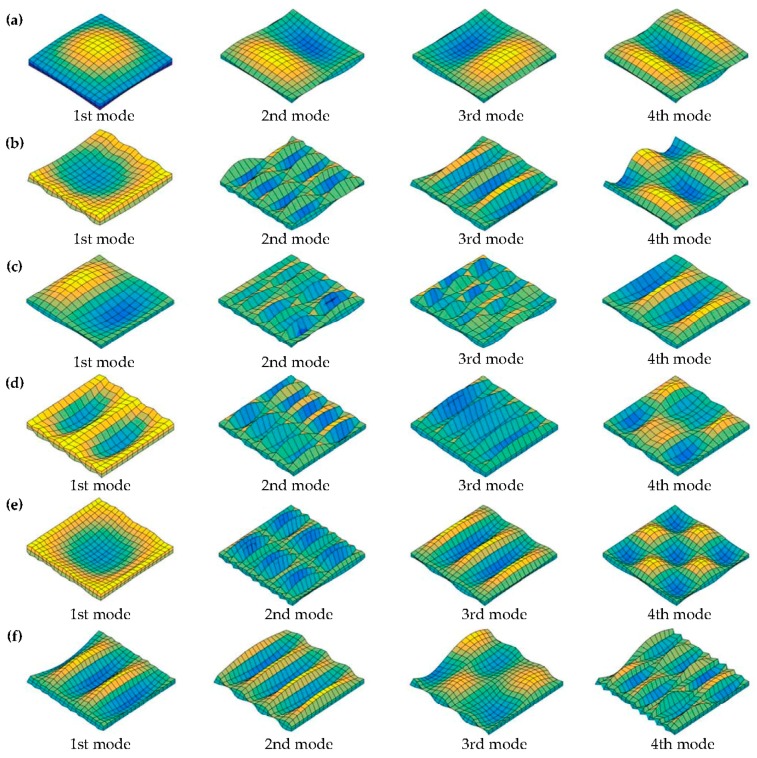
The first four mode shapes of the moderately thick OFG plates with various boundary restraints: (**a**) SSSS; (**b**) SSSF; (**c**) SSSC; (**d**) CFCC; (**e**) CFCS; and (**f**) CFCF.

Numerous new results of fundamental frequencies *f* (Hz) are presented in Table 4 and Table 5 for moderately thick OFG plates with a variety of general boundary restraints. In the case of Table 4 and Table 5, the gradient indexes and geometrical parameters of the OFG plates are taken to be *p* = 0.5, 2, and 10, and *a*/*b* = 1.5 and 2, *h*/*a* = 0.2, 0.3, and 0.5. The boundary restraints, including SSSS, SSSC, SSSF, CFCC, CFCS, CFCF, CFSS and CFSF, are considered. It can be seen from the tables that the solutions of the fundamental frequencies *f* (Hz) corresponding to different boundary restraints have obvious difference. The frequencies of the moderately thick OFG plates with SSSS, SSSC and SSSF are significantly lower the other restraints, this is due to that the smaller restraints at the edges decrease the flexural rigidity of the plate, resulting in smaller frequency response. The frequencies of the OFG plates decrease as thickness–length ratio (*h*/*a*) and length–width ratio (*a*/*b*) increase. The first four mode shapes with SSSS, SSSC, SSSF, CFCC, CFCS and CFCF boundary restraints are depicted in Figure 3 to further enrich the vibration results of OFG plates. The gradient index and geometrical parameters are set as *p* = 1, *a*/*b* = 1.5, and *h*/*a* = 0.3. Next, the effects of gradient index and volume fraction on frequencies in various volume distributions with general boundary restraints are illustrated.

### 3.2. Volume Fraction Analysis

In this study, the material properties are assumed as three types (P-OFG, C-OFG, and S-OFG), which are realized by different ceramic-to-metal volume distributions in the thickness direction. The material properties are assumed to vary through the thickness of the plate with ceramic-to-metal volume distribution between the two surfaces. Specifically, the horizontal and vertical Young’s modulus (*E*_1_ and *E*_2_), density *ρ* and main Poisson ratio $\nu_{12}$ are assumed to vary continuously through the plate thickness.

**Figure 4:**
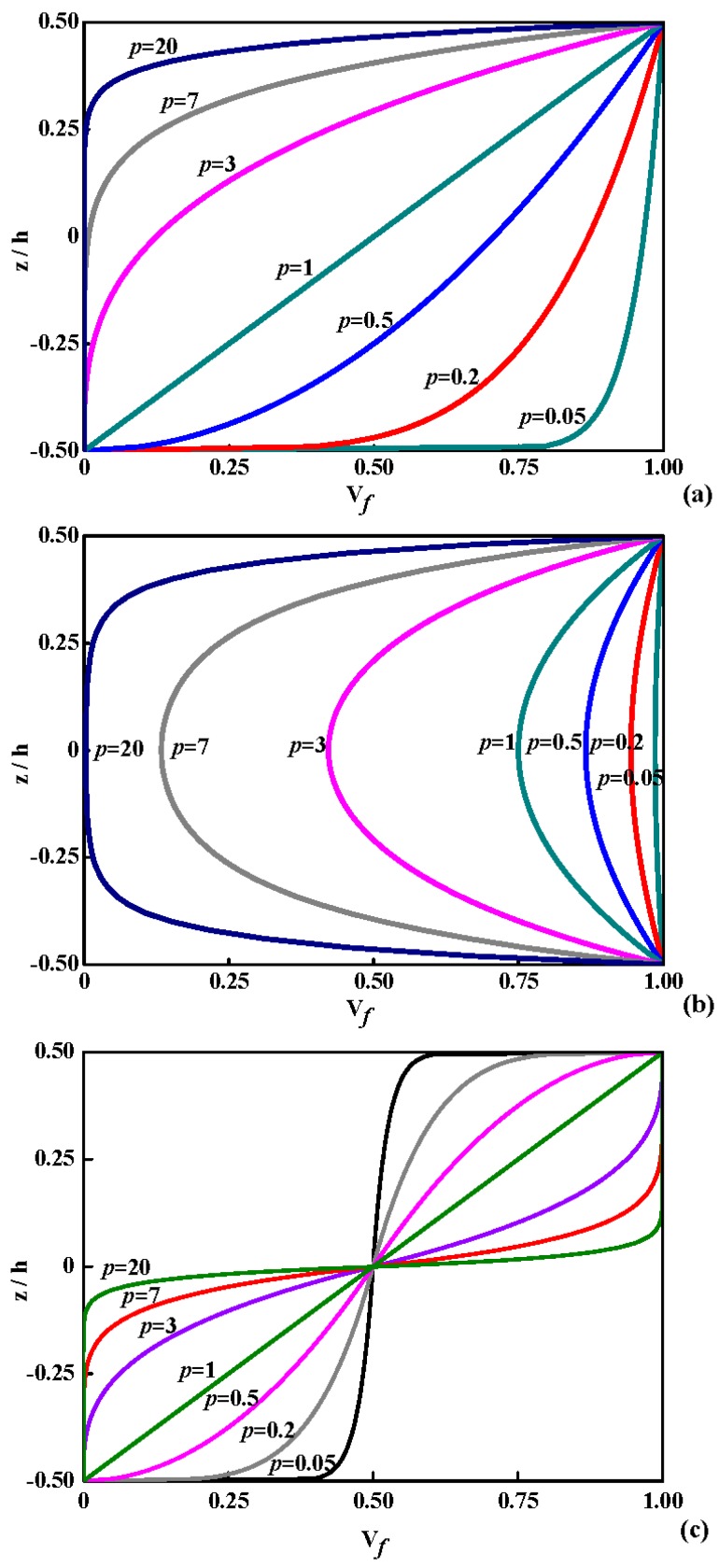
Variation of ceramic volume fraction V*_f_* through the coordinate-thickness ratio in various types: (**a**) P-OFG; (**b**) C-OFG; and (**c**) S-OFG.

According to Equations (1)–(3), the variation of the ceramic volume fraction through coordinate-thickness ratio in various types is presented in Figure 4 (*V*_1_ = 0, *V*_2_ = 1). In Figure 4a, the volume fraction of ceramic varies quickly near the lowest surface for *p* < 1 and increases quickly near the top surface for *p* > 1 under the case of P-OFG. In Figure 4b, the distribution of the ceramic volume fraction is symmetric about the middle surface for C-OFG. In Figure 4c, the distribution is inverse symmetric about the middle surface for S-OFG, and each part is similar to P-OFG.

### 3.3. Fundamental Frequencies Analysis

**Figure 5:**
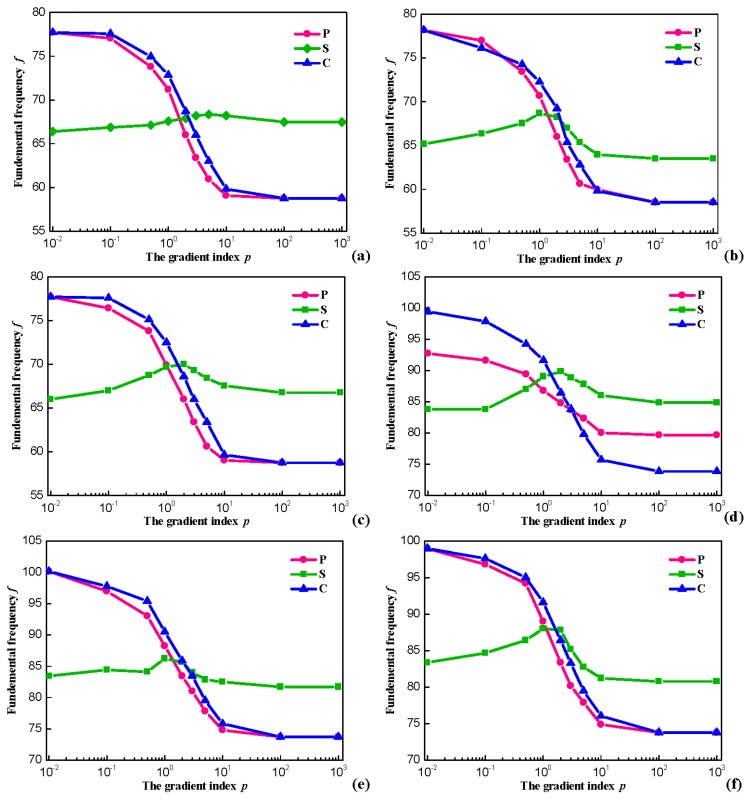
Variation of the frequencies through *p* for OFG plates with *h*/*a* = 0.5: (**a**) OFG plates with SSSS; (**b**) OFG plates with SSSF; (**c**) OFG plates with SSSC; (**d**) OFG plates with CFCC; (**e**) OFG plates with CFCS; and (**f**) OFG plates with CFCF.

In this subsection, the relations between fundamental frequencies *f* (Hz) and gradient index *p* with general boundary restraints for P-, C- and S-OFG plates are contrasted and analyzed. The variation of the fundamental frequencies *f* (Hz) through gradient index *p* for OFG plates with *h*/*a* = 0.3 is shown in Figure 5. SSSS, SSSF, SSSC, CFCC, CFCS and CFCF boundary restraints are studied for P-, C- and S-OFG plates.

The six sets of curves show a similar behavior. For P- and C-OFG, the frequency parameters are considerably decreased by increasing the gradient index *p*. This is because *E_m_* is much smaller than *E_f_* and the stiffness of the plate decreases with the increased distribution range of metal, thus the frequency parameter declines. When the value of *p* equals to zero, a complete ceramic plate is obtained, whereas infinity *p* indicates a complete metal plate. For the case of S-OFG, although there is a small range of fluctuations as *p* changes, the interval of fundamental frequencies *f* (Hz) is not large. Without loss of generality, the ceramic volume fraction always exhibits the opposite changes and the effect of *p* on the stiffness is small. Therefore, *p* has little influence on the fundamental frequencies *f* (Hz) with different boundary restraints overall. 

**Figure 6:**
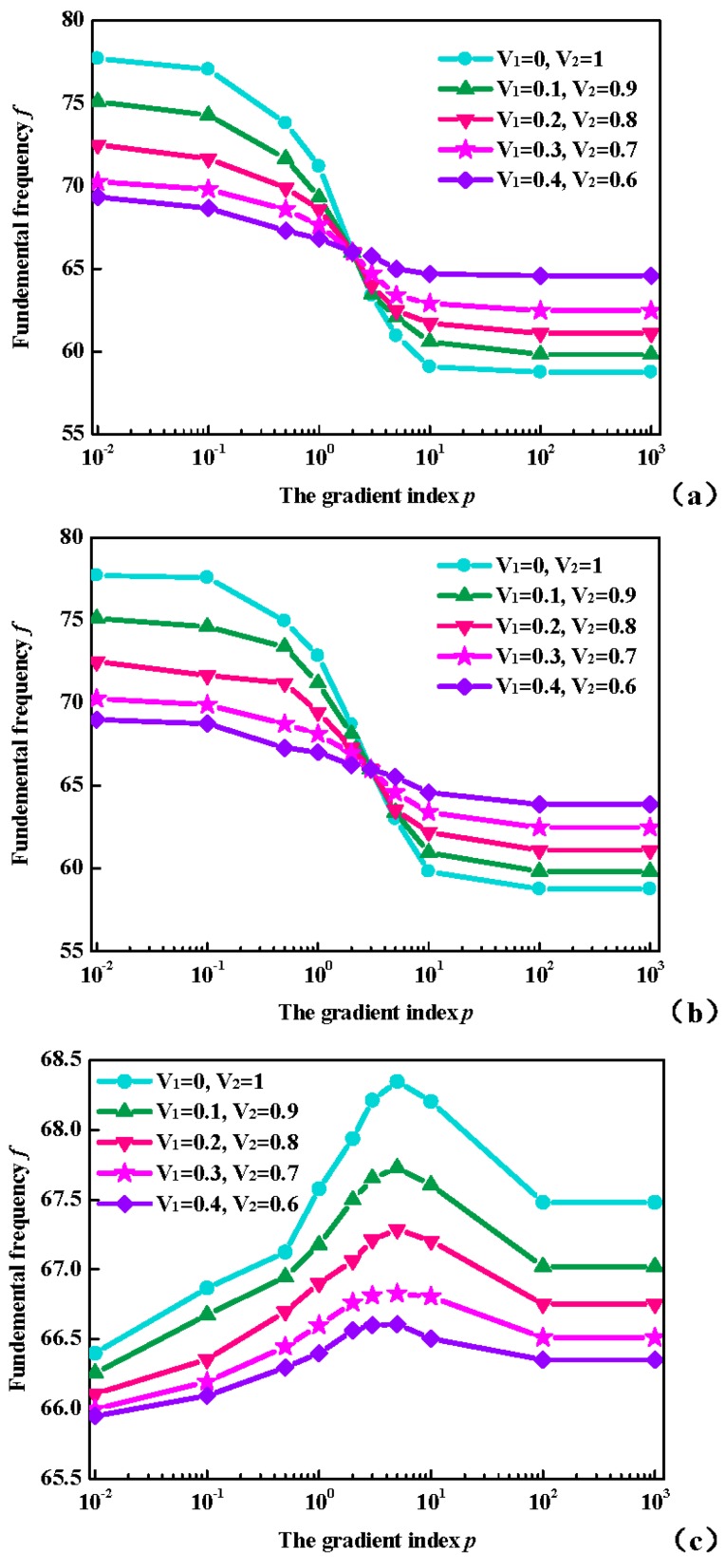
Variation of the fundamental frequencies *f* (Hz) through *p* with simply supported boundary restraint in various types: (**a**) P-OFG; (**b**) C-OFG; and (**c**) S-OFG.

Based on the above analysis, the fundamental frequencies *f* (Hz) for OFG plates with respect to gradient index *p* and volume fractions with simply supported boundary restraint are presented in Figure 6. It can be seen that, with the increase of *V*_2_-*V*_1_, the variation of fundamental frequencies *f* (Hz) through the gradient index *p* is more obvious for OFG plates. The results in Figure 6 show that the effects of the gradient index *p* on the fundamental frequencies *f* (Hz) vary with ceramic volume fractions between bottom and top surfaces.

## 4. Conclusions

In this investigation, a modified Fourier series method has been applied to solve the free vibrations of moderately thick OFG plates with general boundary restraints. Displacements and rotations of each plate, regardless of boundary restraints, are described as an improved form of double Fourier cosine series and several auxiliary functions to effectively eliminate any possible jumps with the original displacement function and its relevant derivatives at the boundaries. Exact solutions are obtained by the energy functions of the plates based on Rayleigh–Ritz method. The general boundary restraints are achieved by setting the stiffness of springs without requiring any special procedures or schemes. It is shown that the present method has high reliability and accuracy. Numerous new results for moderately thick OFG plates with general boundary restraints are presented, which may serve as benchmark solutions for future research in this field.

A comprehensive investigation focused on free vibration properties of OFG materials is given, which serves as a supplement of the properties of FGMs. It is shown that vibration frequencies of the OFG plates are strongly influenced by the ceramic-to-metal volume distribution, gradient index, geometric parameter and boundary restraint. With the interval of ceramic volume fraction increases, the variation of fundamental frequencies through the gradient index is more obvious. When the interval of volume fraction is certain, S-OFG is less affected by the gradient index, while the vibration frequencies of P- and C-OFG are significantly influenced by the gradient index. The effects of the gradient index on the volume fraction are also discussed as well. 

## Figures and Tables

**Table 1:** Main material properties of the used OFG plates.

	Metal (Al)			Ceramic (ZrO_2_)		
Properties	$E_{m}$ (GPa)	$\nu_{m}$	$\rho_{m}$ (kg/m^3^)	$E_{f}$ (GPa)	$\nu_{f}$	$\rho_{f}$ (kg/m^3^)
	70	0.3	2702	200	0.3	5700

**Table 2:** The first five frequency parameters $\Omega =\omega a^{2}\sqrt{\rho h/D}$ of OFG plate with different restraints and thickness–length ratios (*a*/*b* = 0.5).

*h*/*a*	Method	Mode Number				
		1	2	3	4	5
CCCC boundary restraint						
0.1	Present	12.767	13.242	14.454	16.649	19.937
	Ref. [35]	12.767	13.243	14.451	16.647	19.938
0.2	Present	7.5324	8.0879	9.3818	10.206	11.430
	Ref. [35]	7.5325	8.0882	9.3822	10.210	11.435
0.3	Present	5.2981	5.8807	6.8086	7.0848	8.7976
	Ref. [35]	5.2982	5.8807	6.8086	7.0848	8.7975
SSSS boundary restraint						
0.1	Present	8.2283	8.3304	8.8058	10.181	12.615
	Ref. [35]	8.2286	8.3304	8.8058	10.182	12.616
0.2	Present	4.1653	6.0783	6.5920	7.8472	8.3304
	Ref. [35]	4.1652	6.0783	6.5922	7.8472	8.3304
0.3	Present	2.7768	4.6194	5.1094	5.5535	5.5535
	Ref. [35]	2.7768	4.6197	5.1096	5.5536	5.5536
FFFF boundary restraint						
0.1	Present	1.2016	1.6451	3.2673	3.5279	5.8824
	Ref. [35]	1.2016	1.6450	3.2673	3.5278	5.8822
0.2	Present	1.1741	1.5491	3.0822	3.2398	3.9205
	Ref. [35]	1.1742	1.5490	3.0822	3.2398	3.9204
0.3	Present	1.1337	1.4422	2.6131	2.8420	2.9385
	Ref. [35]	1.1337	1.4422	2.6132	2.8421	2.9387

**Table 3:** The comparison of non-dimensional frequency parameters $\varpi =\omega h\sqrt{\rho_{m}/E_{m}}$ for square plates with CCCC, SSSS, CFCF and SCSC boundary restraints, respectively (*h*/*a* = 0.2).

Mode	*p* = 0				*p* = 5				*p* = ∞			
	9 × 9		11 × 11		9 × 9		11 × 11		9 × 9		11 × 11	
	Present	TSDT [15]	Present	TSDT [15]	Present	TSDT [15]	Present	TSDT [15]	Present	TSDT [15]	Present	TSDT [15]
CCCC boundary restraint												
1	0.3598	0.3596	0.3597	0.3598	0.3148	0.3151	0.3154	0.3154	0.3086	0.3090	0.3089	0.3092
2	0.6262	0.6263	0.6282	0.6282	0.5440	0.5441	0.5456	0.5458	0.5379	0.5382	0.5396	0.5398
3	0.6262	0.6263	0.6282	0.6282	0.5440	0.5441	0.5456	0.5458	0.5379	0.5382	0.5396	0.5398
4	0.8462	0.8462	0.8486	0.8486	0.7336	0.7338	0.7357	0.7358	0.7270	0.7272	0.7290	0.7291
5	0.8697	0.8696	0.8687	0.8687	0.7595	0.7597	0.7590	0.7590	0.7471	0.7472	0.7462	0.7464
6	0.8697	0.8696	0.8687	0.8687	0.7595	0.7597	0.7590	0.7590	0.7471	0.7472	0.7462	0.7464
SSSS boundary restraint												
1	0.2463	0.2467	0.2465	0.2462	0.2244	0.2240	0.2236	0.2236	0.2120	0.2120	0.2117	0.2116
2	0.4472	0.4474	0.4485	0.4483	0.3916	0.3914	0.3920	0.3921	0.3846	0.3845	0.3854	0.3852
3	0.4472	0.4476	0.4485	0.4484	0.3916	0.3915	0.3920	0.3922	0.3846	0.3846	0.3854	0.3853
4	0.5405	0.5407	0.5398	0.5397	0.4866	0.4869	0.4862	0.4861	0.4647	0.4646	0.4641	0.4638
5	0.5405	0.5408	0.5398	0.5398	0.4866	0.4869	0.4862	0.4861	0.4647	0.4647	0.4641	0.4638
6	0.6505	0.6509	0.6465	0.6470	0.5691	0.5693	0.5661	0.5659	0.5595	0.5593	0.5562	0.5560
CFCF boundary restraint												
1	0.2379	0.2383	0.2358	0.2362	0.2087	0.2085	0.2101	0.2103	0.2054	0.2055	0.2041	0.2044
2	0.2608	0.2611	0.2611	0.2614	0.2304	0.2300	0.2305	0.2306	0.2230	0.2233	0.2248	0.2250
3	0.4228	0.4231	0.4225	0.4227	0.3672	0.3699	0.3694	0.3696	0.3631	0.3636	0.3629	0.3632
4	0.4250	0.4246	0.4225	0.4248	0.3773	0.3772	0.3765	0.3764	0.3656	0.3649	0.3655	0.3657
5	0.5271	0.5278	0.5307	0.5310	0.4594	0.4592	0.4620	0.4617	0.4543	0.4536	0.4560	0.4563
6	0.5604	0.5603	0.5668	0.5669	0.4902	0.4900	0.4953	0.4952	0.4821	0.4813	0.4869	0.4871
SCSC boundary restraint												
1	0.3068	0.3066	0.3069	0.3066	0.2709	0.2708	0.2710	0.2709	0.2631	0.2635	0.2632	0.2635
2	0.4507	0.4504	0.4510	0.4509	0.3942	0.3940	0.3946	0.3944	0.3868	0.3871	0.3871	0.3875
3	0.5584	0.5579	0.5578	0.5578	0.4932	0.4930	0.4934	0.4930	0.4790	0.4794	0.4790	0.4793
4	0.6068	0.6064	0.6080	0.6082	0.5286	0.5285	0.5305	0.5302	0.5206	0.5211	0.5224	0.5226
5	0.8009	0.8004	0.7995	0.7997	0.6970	0.6967	0.6994	0.6991	0.6872	0.6878	0.6867	0.6871
6	0.8123	0.8118	0.8120	0.8126	0.7096	0.7094	0.7106	0.7101	0.6968	0.6975	0.6978	0.6982

**Table 4:** The first four frequencies *f* (Hz) of moderately thick OFG plates with various boundary restraints, gradient indexes and thickness–length ratios (*a*/*b* = 1.5).

*p*	*h*/*a*	Mode	Boundary Restraints							
			SSSS	SSSC	SSSF	CFCC	CFCS	CFCF	CFSS	CFSF
0.5	0.2	1	149.27	149.27	148.52	183.54	190.92	189.42	182.01	189.43
		2	197.60	197.60	197.79	263.85	251.08	249.90	261.16	249.27
		3	268.70	268.70	267.95	297.59	340.27	341.19	296.66	340.73
		4	283.64	283.64	283.54	360.97	358.36	358.11	363.46	358.11
	0.3	1	73.802	73.08	73.436	89.413	93.036	94.231	89.782	94.232
		2	103.90	103.90	103.31	140.17	131.40	131.22	138.48	131.22
		3	148.69	150.15	149.87	157.01	188.64	189.83	158.70	189.83
		4	158.85	158.85	158.81	200.97	201.05	194.98	201.41	201.98
	0.5	1	22.872	22.871	23.486	25.853	28.699	29.260	26.981	38.988
		2	36.633	36.056	35.439	50.398	46.378	45.522	51.837	45.508
		3	57.082	57.082	56.925	57.403	71.642	71.353	57.263	71.345
		4	60.526	60.526	60.548	75.468	76.557	76.637	75.184	77.240
2	0.2	1	139.82	138.54	137.19	175.35	173.10	173.92	177.47	173.92
		2	176.40	176.40	175.66	218.98	224.11	222.62	219.83	222.62
		3	219.76	220.94	219.01	289.21	277.88	275.82	289.21	279.37
		4	235.79	235.79	235.47	289.58	299.38	300.53	289.34	288.57
	0.3	1	65.998	65.998	65.164	82.771	83.441	83.373	84.466	83.809
		2	89.699	89.699	89.101	112.91	113.12	113.57	112.24	113.57
		3	117.20	117.20	117.80	156.24	148.17	146.97	152.31	146.97
		4	126.79	126.79	128.58	157.38	160.39	160.18	156.24	160.19
	0.5	1	19.385	19.385	19.526	25.401	24.863	24.626	25.130	24.603
		2	29.511	29.510	29.624	37.273	36.784	36.587	37.560	36.582
		3	41.343	41.343	41.060	54.783	52.479	52.129	54.783	52.125
		4	47.861	45.759	45.111	55.233	65.442	57.663	57.311	61.643
10	0.2	1	126.24	126.24	124.07	170.09	159.65	159.72	172.19	159.72
		2	155.05	155.70	155.24	195.56	197.08	197.04	195.59	197.09
		3	181.92	181.92	181.99	234.65	230.57	230.56	235.40	247.57
		4	213.04	195.00	196.87	287.83	248.14	247.57	285.93	268.55
	0.3	1	59.038	59.039	59.980	81.646	72.792	74.173	80.591	74.541
		2	76.164	76.052	77.799	99.184	96.254	96.254	97.498	95.994
		3	91.501	91.798	91.059	122.82	116.05	115.18	121.14	114.86
		4	100.93	100.93	92.095	153.18	126.44	127.23	152.02	127.10
	0.5	1	16.608	16.608	16.210	21.258	20.341	20.629	24.613	20.501
		2	24.392	23.587	23.378	30.328	29.454	29.473	31.454	29.251
		3	28.855	28.855	28.908	42.315	36.206	36.074	41.992	36.187
		4	33.175	33.175	33.255	54.897	41.721	42.218	54.944	42.077

**Table 5:** The first four frequencies *f* (Hz) of moderately thick OFG plates with various boundary restraints, gradient indexes and thickness–length ratios (*a*/*b* = 2).

*p*	*h*/*a*	Mode	Boundary Restraints							
			SSSS	SSSC	SSSF	CFCC	CFCS	CFCF	CFSS	CFSF
0.5	0.2	1	143.27	143.27	142.55	172.14	180.56	180.56	172.96	180.44
		2	169.61	169.61	169.43	217.36	212.08	213.37	215.25	213.37
		3	215.70	215.70	215.71	288.85	268.79	273.19	282.80	275.78
		4	261.46	263.40	261.78	293.42	343.84	318.69	293.74	323.83
	0.3	1	39.957	39.799	39.959	37.882	49.638	50.648	46.905	50.649
		2	50.756	50.756	50.259	66.126	65.693	64.701	64.718	64.685
		3	71.395	71.395	71.466	94.250	88.635	87.408	95.658	89.695
		4	93.545	93.545	94.043	99.961	118.79	118.38	99.716	118.38
	0.5	1	13.742	13.742	13.918	10.946	10.722	12.523	10.099	12.520
		2	21.131	21.130	21.214	17.774	17.161	17.210	18.616	17.424
		3	26.870	29.640	30.154	28.204	26.478	26.538	28.033	26.390
		4	32.248	32.248	31.950	31.325	37.018	36.416	30.482	37.678
2	0.2	1	131.95	131.95	131.92	171.11	167.09	170.62	168.99	167.34
		2	153.97	153.97	153.22	195.56	194.12	193.80	196.22	193.67
		3	189.64	189.64	189.12	234.63	239.11	239.76	234.59	239.76
		4	214.35	214.35	214.52	281.09	271.88	272.33	283.23	273.38
	0.3	1	34.865	34.865	31.766	46.420	44.966	44.964	45.004	41.363
		2	44.032	44.009	35.233	52.573	55.703	56.658	56.257	56.661
		3	59.393	59.393	59.309	73.363	73.826	70.840	73.412	76.400
		4	69.760	69.759	70.258	94.390	88.247	87.151	93.799	87.154
	0.5	1	7.9456	7.9443	8.0661	16.104	10.235	10.082	11.810	10.080
		2	11.244	11.245	11.670	19.577	14.729	14.280	13.584	14.274
		3	16.165	16.522	15.924	27.897	21.322	20.896	20.420	20.891
		4	20.059	19.795	20.059	28.088	26.279	25.679	28.050	25.608
10	0.2	1	121.99	121.78	121.99	164.35	153.67	153.22	164.34	153.22
		2	139.33	139.32	138.89	179.97	177.43	175.93	180.63	175.97
		3	163.88	163.88	164.16	203.80	207.55	208.46	204.18	208.41
		4	176.79	177.01	177.53	234.65	222.20	223.76	235.40	224.36
	0.3	1	26.254	30.634	30.422	43.323	38.697	38.705	43.546	38.856
		2	30.635	38.260	37.712	51.818	47.346	47.606	50.407	47.328
		3	48.066	48.067	48.033	63.056	61.108	58.731	61.700	60.328
		4	51.483	51.482	51.025	74.388	64.981	65.664	74.191	62.648
	0.5	1	6.5105	6.5123	6.6952	12.656	8.0167	8.3461	10.053	7.8646
		2	8.9101	8.9129	9.1915	12.891	10.870	11.210	12.916	11.243
		3	12.426	12.430	12.199	16.145	15.570	15.098	16.666	15.386
		4	12.468	12.468	13.262	21.249	16.497	16.222	20.417	15.884

## Appendix A

Fourier coefficients eigenvector **G**, stiffness matrix **K** and mass matrix **M** of the moderately thick plates:

$$A={\left[A_{00},\cdots ,A_{mn},\cdots ,A_{MN}\right]}^{T}\;a={\left[a_{1}^{0},\cdots ,a_{l}^{n},\cdots ,a_{2}^{N}\right]}^{T}\;b={\left[b_{1}^{0},\cdots ,b_{l}^{m},\cdots ,b_{2}^{M}\right]}^{T}$$

$$B={\left[B_{00},\cdots ,B_{mn},\cdots ,B_{MN}\right]}^{T}\;c={\left[c_{1}^{0},\cdots ,c_{l}^{n},\cdots ,c_{2}^{N}\right]}^{T}\;d={\left[d_{1}^{0},\cdots ,d_{l}^{n},\cdots ,d_{2}^{M}\right]}^{T}$$

$$C={\left[C_{00},\cdots ,C_{mn},\cdots ,C_{MN}\right]}^{T}\;e={\left[e_{1}^{0},\cdots ,e_{l}^{n},\cdots ,e_{2}^{N}\right]}^{T}\;f={\left[f_{1}^{0},\cdots ,f_{l}^{m},\cdots ,f_{2}^{M}\right]}^{T}$$

$$D={\left[D_{00},\cdots ,D_{mn},\cdots ,D_{MN}\right]}^{T}\;g={\left[g_{1}^{0},\cdots ,g_{l}^{n},\cdots ,g_{2}^{N}\right]}^{T}\;h={\left[h_{1}^{0},\cdots ,h_{l}^{m},\cdots ,h_{2}^{M}\right]}^{T}$$

$$E={\left[E_{00},\cdots ,E_{mn},\cdots ,E_{MN}\right]}^{T}\;i={\left[i_{1}^{0},\cdots ,i_{l}^{n},\cdots ,i_{2}^{N}\right]}^{T}\;j={\left[j_{1}^{0},\cdots ,j_{l}^{m},\cdots ,j_{2}^{M}\right]}^{T}$$

The unknown Fourier coefficients eigenvector **G** in the displacement expressions is divided into five parts: $G^{u},G^{v},G^{w},G^{\phi_{x}},G^{\phi_{y}}$.

$$G^{u}={\left[A,\;a,\;b\right]}^{T}=\left[A_{00},A_{01},\cdots ,A_{m0},A_{m1},\cdots A_{mn},\cdots ,A_{MN},a_{1}^{0},\cdots ,a_{l}^{n},\cdots ,a_{2}^{N},b_{1}^{0},\cdots ,b_{l}^{m},\cdots ,b_{2}^{M}\right]$$

$$G^{v}={\left[B,\;c,\;d\right]}^{T}=\left[B_{00},B_{01},\cdots ,B_{m0},B_{m1},\cdots B_{mn},\cdots ,B_{MN},c_{1}^{0},\cdots ,c_{l}^{n},\cdots ,c_{2}^{N},d_{1}^{0},\cdots ,d_{l}^{m},\cdots ,d_{2}^{M}\right]$$

$$G^{w}={\left[C,\;e,\;f\right]}^{T}=\left[C_{00},C_{01},\cdots ,C_{m0},C_{m1},\cdots C_{mn},\cdots ,C_{MN},e_{1}^{0},\cdots ,e_{l}^{n},\cdots ,e_{2}^{N},f_{1}^{0},\cdots ,f_{l}^{m},\cdots ,f_{2}^{M}\right]$$

$$G^{\phi_{x}}={\left[D,\;g,\;h\right]}^{T}=\left[D_{00},D_{01},\cdots ,D_{m0},D_{m1},\cdots D_{mn},\cdots ,D_{MN},g_{1}^{0},\cdots ,g_{l}^{n},\cdots ,g_{2}^{N},h_{1}^{0},\cdots ,h_{l}^{m},\cdots ,h_{2}^{M}\right]$$

$$G^{\phi_{y}}={\left[E,\;i,\;j\right]}^{T}=\left[E_{00},E_{01},\cdots ,E_{m0},E_{m1},\cdots E_{mn},\cdots ,E_{MN},i_{1}^{0},\cdots ,i_{l}^{n},\cdots ,i_{2}^{N},j_{1}^{0},\cdots ,j_{l}^{m},\cdots ,j_{2}^{M}\right]$$

Sub-matrices in the **K** and **M** are listed as follows:

$$H=\left[H_{xy},H_{x},H_{y}\right]$$

$$H_{xy}=\left[\cos\lambda_{0}x\cos\lambda_{0}y,\cdots ,\cos\lambda_{m}x\cos\lambda_{n}y,\cdots ,\cos\lambda_{M}x\cos\lambda_{N}y\right]$$

$$H_{x}=\left[\zeta_{1}^{a}(x)\cos\lambda_{0}y,\cdots ,\zeta_{l}^{a}(x)\cos\lambda_{n}y,\cdots ,\zeta_{2}^{a}(x)\cos\lambda_{N}y\right]$$

$$H_{y}=\left[\zeta_{1}^{b}(y)\cos\lambda_{0}x,\cdots ,\zeta_{l}^{b}(y)\cos\lambda_{m}x,\cdots ,\zeta_{2}^{b}(y)\cos\lambda_{M}x\right]$$

$$\begin{array}{cc} \left\{K_{uu}\right\} & =\int_{0}^{a}\int_{0}^{b}\left\{A_{11}\frac{\partial H^{T}}{\partial x}\frac{\partial H}{\partial x}+A_{66}\frac{\partial H^{T}}{\partial y}\frac{\partial H}{\partial y}\right\}dydx+\int_{0}^{b}\left\{k_{x_{0}}^{u}H^{T}H|{}_{x=0}+k_{x_{1}}^{u}H^{T}H|{}_{x=a}\right\}dy\\ & +\int_{0}^{a}\left\{k_{y_{0}}^{u}H^{T}H|{}_{y=0}+k_{y_{1}}^{u}H^{T}H|{}_{y=b}\right\}dx \end{array}$$

$$\begin{array}{cc} \left\{K_{vv}\right\} & =\int_{0}^{a}\int_{0}^{b}\left\{A_{22}\frac{\partial H^{T}}{\partial y}\frac{\partial H}{\partial y}+A_{66}\frac{\partial H^{T}}{\partial x}\frac{\partial H}{\partial x}\right\}dydx+\int_{0}^{b}\left\{k_{x_{0}}^{v}H^{T}H|{}_{x=0}+k_{x_{1}}^{v}H^{T}H|{}_{x=a}\right\}dy\\ & +\int_{0}^{a}\left\{k_{y_{0}}^{v}H^{T}H|{}_{y=0}+k_{y_{1}}^{v}H^{T}H|{}_{y=b}\right\}dx \end{array}$$

$$\begin{array}{cc} \left\{K_{ww}\right\} & =\int_{0}^{a}\int_{0}^{b}\left\{A_{44}\frac{\partial H^{T}}{\partial y}\frac{\partial H}{\partial y}+A_{55}\frac{\partial H^{T}}{\partial x}\frac{\partial H}{\partial x}\right\}dydx+\int_{0}^{b}\left\{k_{x_{0}}^{w}H^{T}H|{}_{x=0}+k_{x_{1}}^{w}H^{T}H|{}_{x=a}\right\}dy\\ & +\int_{0}^{a}\left\{k_{y_{0}}^{w}H^{T}H|{}_{y=0}+k_{y_{1}}^{w}H^{T}H|{}_{y=b}\right\}dx \end{array}$$

$$\begin{array}{cc} \left\{K_{\phi_{x}\phi_{x}}\right\} & =\int_{0}^{a}\int_{0}^{b}\left\{D_{11}\frac{\partial H^{T}}{\partial \phi_{x}}\frac{\partial H}{\partial \phi_{x}}+D_{66}\frac{\partial H^{T}}{\partial y}\frac{\partial H}{\partial y}+A_{55}H^{T}H\right\}dydx+\int_{0}^{b}\left\{k_{x_{0}}^{x}H^{T}H|{}_{x=0}+k_{x_{1}}^{x}H^{T}H|{}_{x=a}\right\}dy\\ & +\int_{0}^{a}\left\{k_{y_{0}}^{x}H^{T}H|{}_{y=0}+k_{y_{1}}^{x}H^{T}H|{}_{y=b}\right\}dx \end{array}$$

$$\begin{array}{cc} \left\{K_{\phi_{y}\phi_{y}}\right\} & =\int_{0}^{a}\int_{0}^{b}\left\{D_{22}\frac{\partial H^{T}}{\partial \phi_{y}}\frac{\partial H}{\partial \phi_{y}}+D_{66}\frac{\partial H^{T}}{\partial x}\frac{\partial H}{\partial x}+A_{44}H^{T}H\right\}dydx+\int_{0}^{b}\left\{k_{x_{0}}^{y}H^{T}H|{}_{x=0}+k_{x_{1}}^{y}H^{T}H|{}_{x=a}\right\}dy\\ & +\int_{0}^{a}\left\{k_{y_{0}}^{y}H^{T}H|{}_{y=0}+k_{y_{1}}^{y}H^{T}H|{}_{y=b}\right\}dx \end{array}$$

$$\left\{K_{uv}\right\}=\int_{0}^{a}\int_{0}^{b}\left\{A_{12}\frac{\partial H^{T}}{\partial x}\frac{\partial H}{\partial y}+A_{66}\frac{\partial H^{T}}{\partial y}\frac{\partial H}{\partial x}\right\}dydx$$

$$\left\{K_{u\phi_{x}}\right\}=\int_{0}^{a}\int_{0}^{b}\left\{B_{11}\frac{\partial H^{T}}{\partial \phi_{x}}\frac{\partial H}{\partial \phi_{x}}+B_{66}\frac{\partial H^{T}}{\partial y}\frac{\partial H}{\partial y}\right\}dydx$$

$$\left\{K_{u\phi_{y}}\right\}=\int_{0}^{a}\int_{0}^{b}\left\{B_{12}\frac{\partial H^{T}}{\partial x}\frac{\partial H}{\partial \phi_{y}}+B_{66}\frac{\partial H^{T}}{\partial \phi_{y}}\frac{\partial H}{\partial x}\right\}dydx$$

$$\left\{K_{v\phi_{x}}\right\}=\int_{0}^{a}\int_{0}^{b}\left\{B_{12}\frac{\partial H^{T}}{\partial y}\frac{\partial H}{\partial \phi_{x}}+B_{66}\frac{\partial H^{T}}{\partial \phi_{x}}\frac{\partial H}{\partial y}\right\}dydx$$

$$\left\{K_{v\phi_{y}}\right\}=\int_{0}^{a}\int_{0}^{b}\left\{B_{22}\frac{\partial H^{T}}{\partial \phi_{y}}\frac{\partial H}{\partial \phi_{y}}+B_{66}\frac{\partial H^{T}}{\partial x}\frac{\partial H}{\partial x}\right\}dydx$$

$$\left\{K_{\phi_{x}\phi_{y}}\right\}=\int_{0}^{a}\int_{0}^{b}\left\{D_{12}\frac{\partial H^{T}}{\partial \phi_{x}}\frac{\partial H}{\partial \phi_{y}}+D_{66}\frac{\partial H^{T}}{\partial \phi_{y}}\frac{\partial H}{\partial \phi_{x}}\right\}dydx$$

$$\left\{K_{w\phi_{x}}\right\}=\int_{0}^{a}\int_{0}^{b}\left\{A_{55}\frac{\partial H^{T}}{\partial \phi_{x}}H\right\}dydx$$

$$\left\{K_{w\phi_{y}}\right\}=\int_{0}^{a}\int_{0}^{b}\left\{A_{44}\frac{\partial H^{T}}{\partial \phi_{y}}H\right\}dydx$$

$$\left\{K_{uw}\right\}=0$$

$$\left\{K_{vw}\right\}=0$$

$$M_{uu}=M_{vv}=M_{ww}=\int_{0}^{a}\int_{0}^{b}I_{0}H^{T}Hdydx$$

$$M_{u\phi_{x}}=M_{v\phi_{y}}=\int_{0}^{a}\int_{0}^{b}I_{1}H^{T}Hdydx$$

$$M_{\phi_{x}\phi_{x}}=M_{\phi_{y}\phi_{y}}=\int_{0}^{a}\int_{0}^{b}I_{2}H^{T}Hdydx$$
